# Improvements in GROMACS plugin for PyMOL including implicit solvent simulations and displaying results of PCA analysis

**DOI:** 10.1007/s00894-016-2982-4

**Published:** 2016-04-23

**Authors:** Tomasz Makarewicz, Rajmund Kaźmierkiewicz

**Affiliations:** Laboratory of Biomolecular Systems Simulations, Intercollegiate Faculty of Biotechnology, University of Gdańsk and Medical University of Gdańsk, Abrahama 58, 80-307 Gdańsk, Poland

**Keywords:** Dynamics, GROMACS, Plugin, PyMOL

## Abstract

In order to get the dynamic molecule model from the static one, the molecular dynamics (MD) simulation needs to be performed. Some software sets such as GROMACS are used for that purpose. Unfortunately they lack GUI. The Dynamics PyMOL plugin allows researcher to perform MD simulations directly from the PyMOL software by GUI-based interface of GROMACS tools. This paper describes many improvements introduced into the Dynamics PyMOL plugin 2.0 including: an integration with ProDy library, possibility to use the implicit solvents, an ability to interpret the MD simulations, and implementation of some more GROMACS functionality.

## Introduction

Structures of molecules obtained from X-ray crystallography [1], nuclear magnetic resonance [2], and other methods [3] are stored in the Protein Data Bank [4, 5]. They are formatted as PDB files [6] and one of the most popular software to visualize them is PyMOL [7]. Biomolecules in the real world demonstrate dynamics behavior, so it is not enough, to only analyze the static PDB file in order to obtain full knowledge of molecular properties. One of the software that allows molecular dynamics (MD) simulations is GROMACS [8–12]. PyMOL exposes an API for third party plugins [13], we took advantage of that fact and the Dynamics PyMOL plugin [14] was developed to facilitate molecular dynamics simulations in PyMOL GUI, using the underlying GROMACS.

Performing molecular dynamics simulation is a relatively complicated task, therefore every option to reduce level of complexity of calculations is welcome. One of such options is the reduction of the number of interacting centers through the use of an implicit solvent model. Although the explicit solvent is still the standard solution model in molecular dynamics simulations and GROMACS offers TIP3P, TIP4P, SPC, and their variants as a water model [15], the calculation using thousands of those water molecules is computationally demanding, as they interact not only with the molecule of interest, but with each other as well. The system with explicit solvent needs to be placed into the so-called water box. The water box, if used without the proper periodic boundary conditions, would lead to undesired border effects, which could disturb the whole simulation. Those limitations were stimulus to develop other methods of handling solvent in molecular dynamics simulations. GROMACS offers implicit solvent as an alternative and exposes three generalized Born (GB) implementations: Still, Hawkins-Cramer-Truhlar (HCT), Onufriev-Bashford-Case (OBC) [16]. Those implementations offer significant time reduction for molecular dynamics calculations and also the reduction of system complexity makes it easier to avoid the risk of simulation failure [17].

The results of the molecular dynamics simulation can be visualized as an animation. Those animations give researchers important information about movement of the molecule, but usually they are not enough. In order to obtain more data from the system it is critical to perform proper analysis of the MD results. The correct interpretation of MD data can produce static images presenting the results of dynamic behavior of the molecule shown in the form of, for example, vectors pointing into direction of the highest displacement of the molecular fragment. It also provides quantitative interpretation of the results, which can be used for comparison with other simulations or for further analysis. Two methods are particularly useful in aiding the interpretation of MD results, these are the normal mode analysis (NMA) and the principal component analysis (PCA).

The normal mode analysis method has been successfully used [18] to determine and investigate the approximate protein dynamics. It models protein or other molecules as a harmonic oscillating system and classifies all possible deformations around a stable equilibrium. The local deformations are represented by high frequency modes, while larger movements are represented by low frequency modes. The all-atoms NMA (aaNMA) is computationally demanding and therefore is limited to systems containing hundreds of residues. The common solution overcoming this limitation is to reduce the number of degrees of freedom, which can be achieved by, fixing lengths and angles of bonds in the molecule or by calculating only rotation and translation of some residues [19]. Using those approximations enables application of aaNMA to simulations of a protein molecules consisting of a few thousand residues. Another variant of NMA is an elastic network model (ENM), in which the mechanics defined by the empirical force field is replaced by a ball and spring harmonic potential [20]. This method can be combined with a coarse grained representation of the protein molecule. In such a situation, usually, only C^α^ atoms are considered. The method is simple, efficient, and provides reliable results, therefore, an ENM method has emerged as the preferred approach to perform NMA on proteins containing several thousands of residues [21]. One of the most popular variants of ENM is an anisotropic network model (ANM) [21].

Another approach to analyze the protein dynamics is to use the principal component analysis method [22]. The results of molecular dynamics simulation (or, equivalently, the large set of experimental structures of a single protein) can be used as an input for PCA to create principal components (PCs), which are then sorted in accordance to their contribution to the total fluctuation of a protein molecule. Only a small part of these PCs describe a great majority of the whole atomic movement [23]. Those selected PCs are the foundation of the essential dynamics (ED) and are most interesting for researchers when studying conformational changes of the proteins.

One of the new and promising software for protein structural dynamics analysis is ProDy [24]. It allows performing both ANM and PCA. ProDy has fast and flexible PDB and DCD file parsers, and powerful and customizable atom selections for contact identification and structure comparisons. It provides API for third party software to utilize its capabilities. The results can be visualized using NMWiz, which is the plugin for VMD software. Despite being rather new software in the field, it had already found the use in: the normal mode analysis of replication protein A (RPA) interactions [25], searching for the correlations between protein sequence evolution and its dynamics [26], quantifying the structural flexibility of Vps32 protein [27], and NMA of selected *Yersinia enterocolitica* proteins [28].

## Methods

The Dynamics PyMOL plugin is written in Python [29] programming language. Therefore, the code is clear, easy to understand and modify by researchers. Version 2.7 [30] of this programming language is used by the software, just like in the PyMOL case [31]. TkInter [32] is used for displaying graphics both in plugin and PyMOL [31]. To summarize, the plugin is as compatible with PyMOL as it is possible and uses the same system libraries and the same version of programming language as PyMOL to keep additional dependencies minimal.

In order to perform the molecular dynamics calculation, the GROMACS package is needed. The designed plugin is now compatible with the latest GROMACS 5.0 [33], but can also work with the previous versions of this software (for example 4.6). The ProDy [24] library is an optional dependency. It is an open-source package for protein structural dynamics analysis. It has Python API which is utilized for advanced dynamics interpretation.

The plugin is designed for Unix-like systems and it requires Linux, Unix or Mac OS X in order to run.

The plugin can be installed by PyMOL built-in plugin installation tool (menu bar- > Plugin- > Manage Plugins- > Install…) and after this procedure is added to the Plugin submenu in the menu bar. On Ubuntu Linux the latest stable version of the plugin is also available by the PPA repository [34] and can be installed by simple, standard command “sudo apt-get install pymol-plugin-dynamics”.

## Results

### The plugin’s workflow

**Fig. 1:**
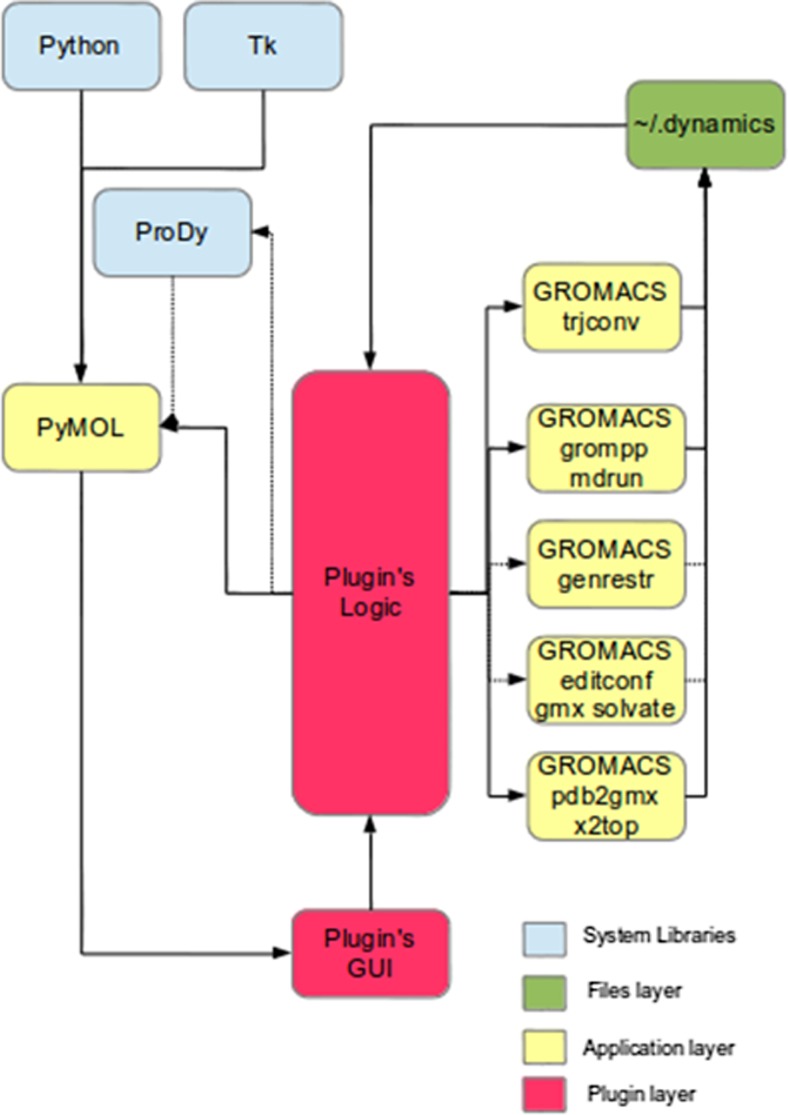
The workflow of communication between PyMOL and GROMACS modules using authors plugin

The Fig. 1 shows the workflow of Dynamics PyMOL plugin. At first a protein structure file (usually a PDB file) [6] is loaded into PyMOL. Once the plugin is started it will allow the user to select one of the PyMOL loaded protein models or select any other arbitrary PDB file. Then the user needs to choose the specific simulation conditions using the provided GUI and press OK to start the molecular dynamics calculations. First the GROMACS pdb2gmx [35] or x2top [36] tool will be started to convert the PDB formatted file into the format required by GROMACS, which is suitable for further calculations. Then, if an explicit solvent simulation is requested, the editconf [37] and gmx solvate [38] tools will add a water box around the solute molecule using the chosen water model. This step will be omitted if an implicit solvent is being used, as in such a situation, the water box is not required [33]. After that Grompp [39] and mdrun [40] GROMACS tools will perform minimization of energy, olecular dynamics (MD) simulation with positional restrains and the final molecular dynamics simulation. The GROMACS tool genrestr [41] will handle restraints, if they were selected. Finally, the trjconv [42] tool will convert results into a multimodel PDB file, which will be displayed in PyMOL viewer. If the ProDy library is present in the system, then additional calculations will be made and their results will be displayed in PyMOL. An additional window will pop up, which will allow advanced dynamics interpretation. All files, configurations, and progress status are stored and processed in the ~/.dynamics directory.

The Dynamics PyMOL plugin can be started by clicking on top PyMOL menu bar Plugin- > dynamics. The software is meant to be as easy to use as possible. It takes as an input a PDB formatted molecule model and it creates PDB multimodel molecule file as an output, which could be then easily stored and viewed.

### Integration with ProDy

**Fig. 2:**
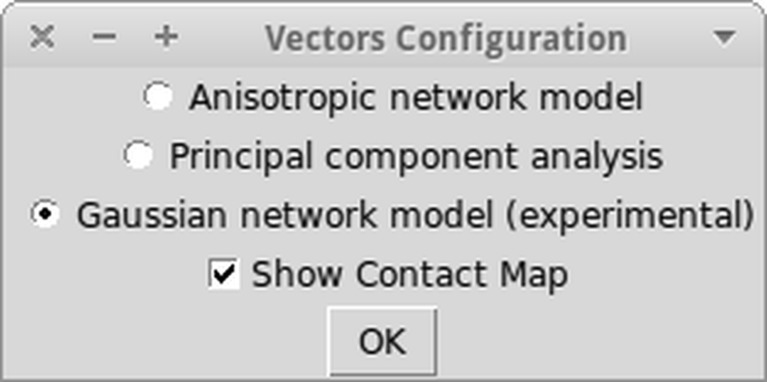
Implemented ProDy options for displaying vectors

The most important improvement, which was introduced to Dynamics PyMOL plugin is integration with ProDy library. It is fully optional and lack of this library neither will prevent the plugin from working, nor cripple any of its other functionality. ProDy allows the researcher to calculate the normal modes, which can be used afterward to display results of molecular dynamics simulation as vectors, the resulting images are sometimes called “the porcupine plots”. It also allows the researcher to calculate the Kirchoff matrix, which then can be used to display contact Map and cross-correlations. Figure 2 shows ProDy configuration options included in the plugin. To get the final results an Anisotropic network model (ANM) [21], Gaussian network model (GNM) [43] or principal component analysis (PCA) [22] can be selected. Additionally, the option for showing the contact map on the PyMOL viewer can be selected.

**Fig. 3:**
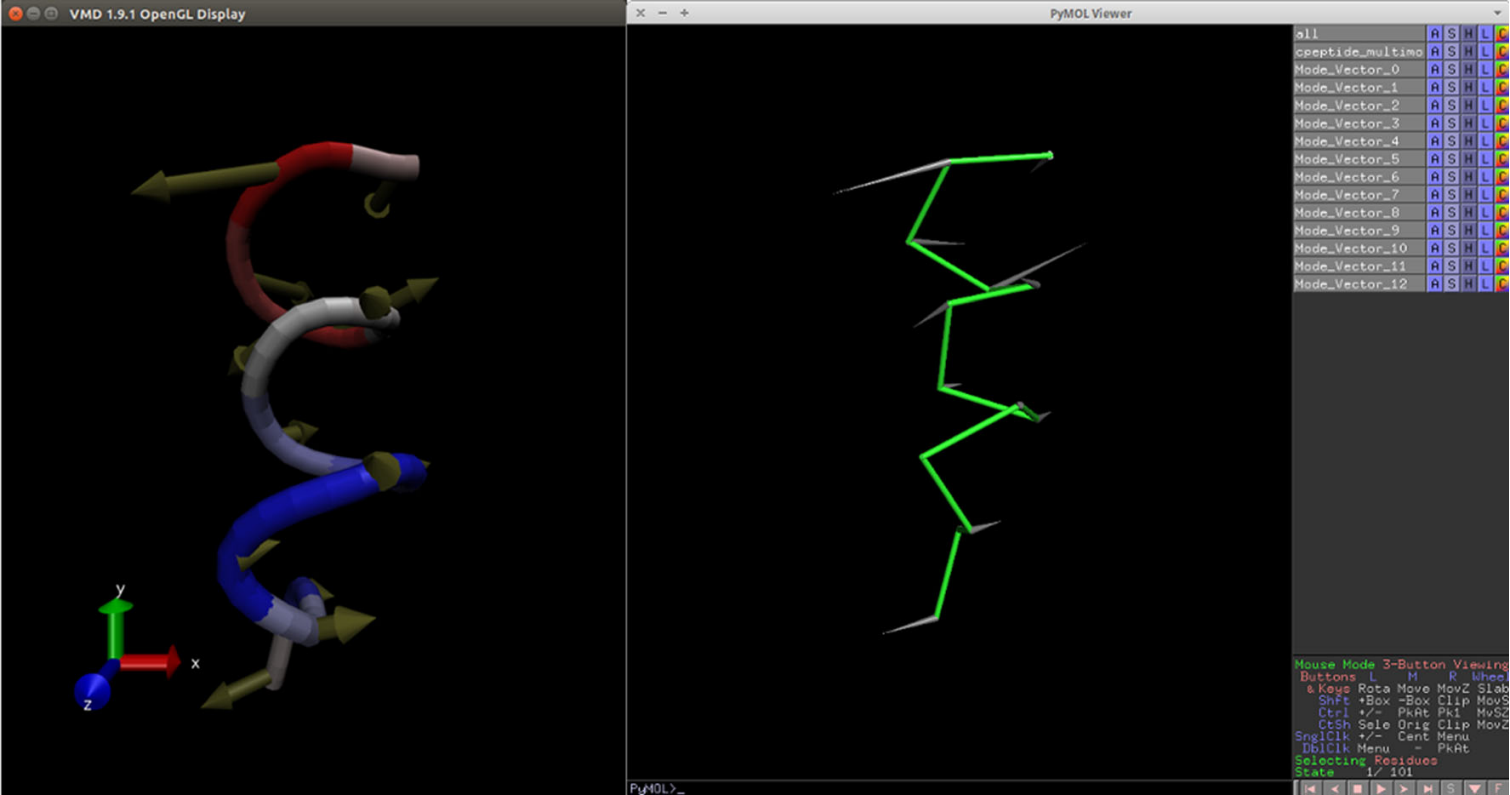
Comparison of the same nmd file opening on VMD (*left*) and PyMOL with the plugin (*right*)

ProDy is the python library meant to be used from command line. It saves results in the nmd format [44]. Normal Mode Wizard (NMWiz) plugin [45] for VMD software [46] used to be the only way to display results of ProDy calculations. With the integration of ProDy, the Dynamics PyMOL plugin also allows viewing nmd files. Figure 3 shows the same nmd file viewed in VMD and PyMOL using the Dynamics PyMOL plugin.

**Fig. 4:**
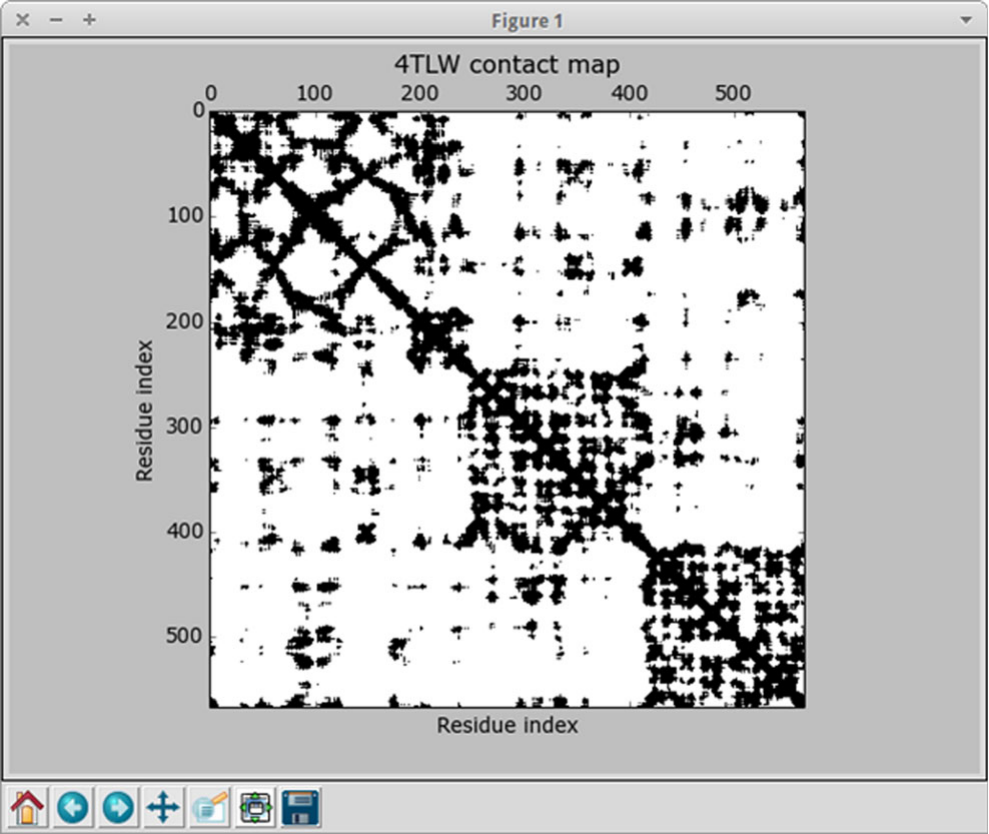
Sample contact map calculated by ProDy

Integration with ProDy enables also a few additional options in the molecular dynamics interpretation window, including possibility to display the Contact Map and Cross-correlations. Figure 4 shows the sample Contact Map.

### Implicit solvents

**Fig. 5:**
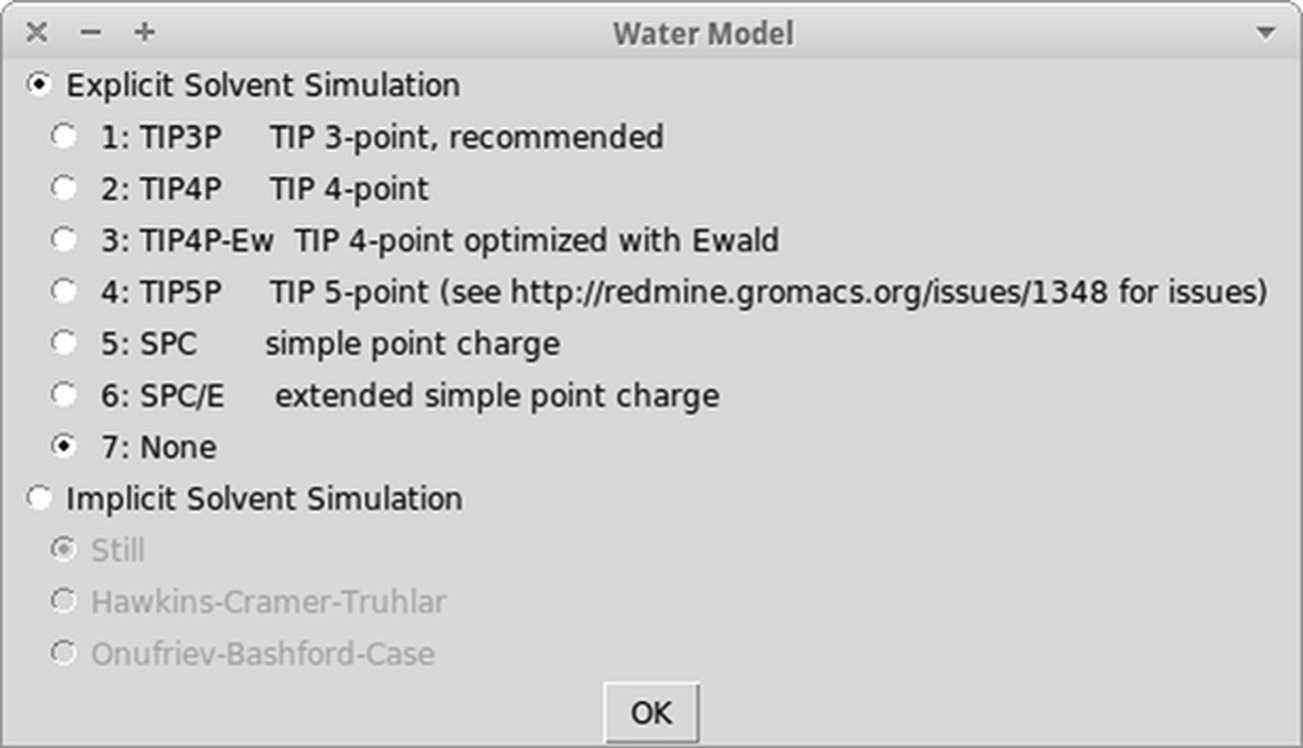
The water model configuration window

Implicit solvation is a feature introduced to GROMACS to lower compute power needed for performing the molecular dynamics simulations [17]. In contrast to the default explicit solvent, it treats the solvent model not as a collection of molecules, but as continuous matter. It is less accurate, when compared to the reality, but allows reduction of calculations needed to perform molecular dynamics simulation. When implicit solvent is selected, then, during calculations, adding the water box step is omitted. There is also no need for positional restrains MD which is automatically omitted. The additional advantage is, that the other calculation steps take less time, as the complexity of the system is significantly reduced. Using an implicit solvent makes results less accurate, but this sacrifice of accuracy, in most cases, is really low. Moreover, this technique removes the possibility of big mistakes, such as accidental omission of border conditions of the water box, it also makes configuration of the proper molecular dynamics system easier. Figure 5 shows the water model configuration window, which allows selection of an implicit solvent model.

### Interpretation of molecular dynamics

**Fig. 6:**
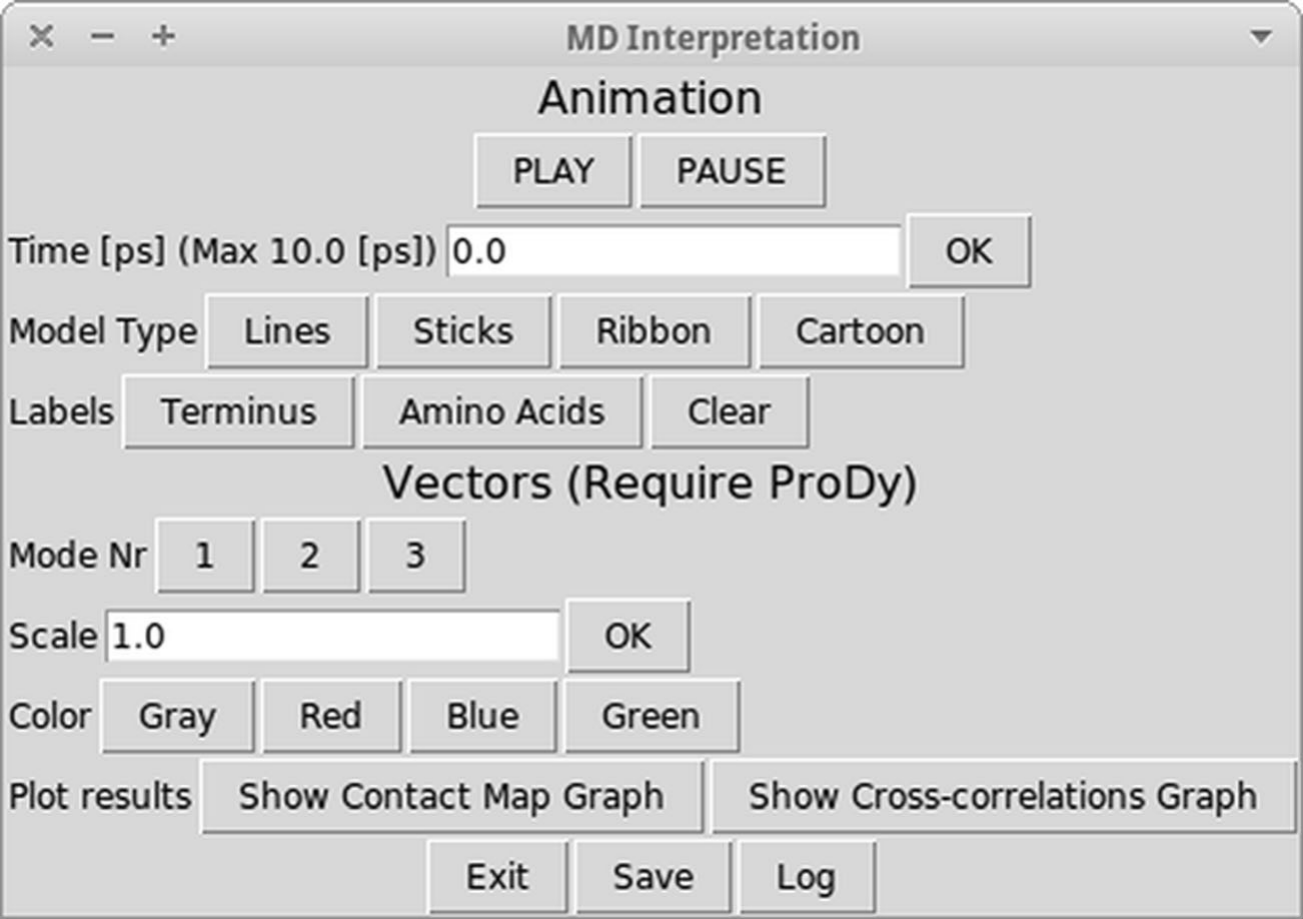
The dynamics interpretation window

Another big improvement to the Dynamics PyMOL plugin is the introduction of a molecular dynamics interpretation window displayed after all calculations are finished. Figure 6 shows this feature. This window is presented together with the results of molecular dynamics simulation in the PyMOL viewer. It includes a set of options, which allow the researcher to manipulate MD results in such a way, that he or she can obtain as much visual data, as is desired. The researcher can start and stop displaying the animation of molecular dynamics, he or she can also switch to the chosen frame of the animation, which corresponds to the specific picosecond of actual MD simulation. The visualization of the model can be altered by selecting lines, sticks, ribbon or cartoon as a model type representation. The terminal amino acid residues or the whole amino acid sequence can be labeled by three letter shortcuts. There are also additional options, which can be used when the ProDy library is present. If so, then the researcher can choose one of the normal modes calculated by ProDy, which will be displayed in the form of vectors, he or she can choose the scale of those vectors and change their colors. Finally, if the contact map option was selected in ProDy configuration, then both contact map and cross-correlation can be displayed. Figure 4 shows a sample contact map, which can be displayed by clicking the appropriate button in the molecular dynamics interpretation window.

### Other GROMACS functionality

There are many other smaller improvements introduced to the software to date, which extends its capabilities and hopefully makes it more user friendly. First of all, until now, only the GROMACS pdb2gmx [35] was used to transform PyMOL loaded PDB file into GROMACS format. However, the pdb2gmx cannot handle residues which are not included in the residue database for a given force field [47]. Now, the plugin offers an option to transform the input file to GROMACS format using x2top [36]. It allows conversion of an arbitrary PDB file to GROMACS format as long as appropriate n2t files are present for atom type translation [48].

The time of molecular dynamic simulation is one of the most important factors in visualization of its results. In accordance to GROMACS specification it can be set by changing the value of time step for integration (dt) and the number of steps (nsteps) in molecular dynamics options [49]. The additional field is now added in the main window. It allows specifying the time of molecular dynamics simulation in picoseconds. Changing this value will automatically alter the number of nsteps to achieve the desired time of simulation.

There are also some improvements in the water box default options. The water box shape (editconf -bt) is now, by default, a dodecahedron instead of cubic. The volume of a dodecahedron, with the same periodic distance, is 0.71 times that of the cube [37], which significantly reduces compute power required for the entire simulation. The water box was also enlarged, by default, to 0.6 value (editconf -d), which allows avoiding undesired border conditions in more cases. All of those values of course can be changed by users through the water box Options window.

Setting the correct molecular dynamics conditions may be of a “trial and error” nature. If the GROMACS software encounters an error during the process, the simulation will stop and the error message will be displayed in the new window. The new feature, which is now introduced, is a LOG button present in the calculation and interpretation window. It will display the full GROMACS output and therefore it allows the researcher to examine all warnings and errors and assist in solving the issues with improperly set dynamics conditions. This functionality is especially useful if no fatal error was encountered, but still simulation did not proceed as expected and the researcher wishes to analyze the cause.

We polished the plugin to be more user friendly, the pop-up balloons with some explanations will now appear after hovering a mouse cursor over an option or stopping cursor movement for some time.

With the support of Mac OS X users [50], the plugin now works fine on OS X platform, if the rest of the stack is present in the system (i.e., when the PyMOL and GROMACS programs are installed).

The scroll bars and progress bars where ported from Tix to ttk, which removes Tix from plugin dependencies and therefore make the plugin easier to install (the ttk is a part of standard Python installation).

## Conclusions

The Dynamics PyMOL plugin was enhanced with tons of features including: displaying the results of MD with vectors, options to use implicit solvents, window supporting an interpretation of MD, ability to use x2top as an optional GROMACS tool, easy set up of the desired length of simulation, water box optimization, better GROMACS error and warning handling, support for Mac OS X, removing Tix dependence. With all these changes the software is marked as version 2.0. The plugin is now much more useful and provides easier and better solutions for many molecular dynamics simulation use scenarios. The software remains to be open source like GROMACS and PyMOL and provides a full open source set of utilities for viewing, setting, and performing molecular dynamics simulation using only the easy GUI tools. This allows the researcher to obtain all of those programs free of charge and modify them as he or she wishes. The source code of the plugin is available under the terms of GPLv3 license from https://github.com/tomaszmakarewicz/Dynamics. The Ubuntu Linux users can install it from official PPA repository: https://launchpad.net/~tomaszm/+archive/dynamics.

## Discussion

The dynamics PyMOL plugin version 2.0 is an important step toward easy and yet powerful molecular dynamics simulation using GUI. It introduces many features, which allows for more comprehensive use of GROMACS software and also introduces additional capabilities offered by ProDy. Nevertheless, researchers need to remember that performing molecular dynamics simulation is a complex work and in many cases the default plugin settings will not produce the proper results. One could encounter errors and familiarization with GROMACS manual may be required in order to set dynamics conditions properly. This plugin should work correctly on the wide range of Unix and Unix-like operating systems as long as dependency software is installed, but Microsoft Windows operating system is not yet supported.

The software is open source and free of charge, which gives researchers easy access to it. On the developer side the feedback from users allows correction of bugs and introduction of new requested features. The plugin is constantly improving and in the future it should incorporate new GROMACS capabilities and still make them as easy to use as possible.
